# Association of Tear and Serum Proteomic Profile on Disease Severity in Vernal Keratoconjunctivitis (VKC)

**DOI:** 10.22336/rjo.2026.56

**Published:** 2026

**Authors:** Supriya Dhar, Amit Gupta, Barkha Gupta, Chintan Malhotra, Arun Kumar Jain, Yashwant Kumar

**Affiliations:** 1 Department of Ophthalmology, Army Hospital RR, Delhi Cantt, New Delhi, India; 2 Advanced Eye Centre, PGIMER, Chandigarh, India; 3 Consultant Ophthalmology, The Healing Touch Superspecialty Eye Care Hospital, New Delhi, India; 4 Department of Immunopathology, PGIMER, Chandigarh, India

**Keywords:** cytokines, inflammatory mediators, serum, tear allergens, tears, VKC, VKC = Vernal keratoconjunctivitis, TIMP = Tissue Inhibitor of Metalloproteinase, IL = Interleukin, TNF = Tumor Necrosis Factor, IFN = Interferon, IgE = Immunoglobulin E, OSDI = Ocular surface Disease Index, TBUT = Tear Break Up Time, PBS = Phosphate Buffered Saline, ELISA = Enzyme Linked Immunosorbent Assay, CBA = Cytokine Bead Analysis, MMP = Matrix metalloproteinase

## Abstract

**Objective:**

To study the association of tear and serum proteomic profile with disease severity in vernal keratoconjunctivitis (VKC).

**Methods:**

A prospective, non-randomized, observational study. 50 participants aged 8-16 years were included in the study: 10 Controls (Group 1), 20 in Moderate VKC (Group 2), and 20 in severe VKC (Group 3). The more inflamed eye was selected for tear sampling. Flow cytometry quantified tear and serum cytokines; TIMP1,2, total IgE, and Vitamin D were studied.

The Primary outcome measures assessed the correlation between disease severity and tear levels, serum IL-2-10,17, TNF-α, IFN-γ, TNF-α, IFN-α, serum TIMP-1,2, IgE, tear allergen assay, and tear-specific IgE in enrolled patients. Secondary outcome measures assessed disease severity with keratoconus and ocular surface disease index (OSDI).

**Results:**

An overall increase in inflammatory mediators in tears was observed in Group 2 compared to Group 1. Higher IL-6 levels were noted in tear samples in groups 2 and 3. Serum IL-10 was elevated in controls compared to diseased patients (p=0.086). Serum IL-17A was detected only in patients with active disease. Keratoconus was present in 15% and 50% of subjects in Groups 2 and 3, respectively (p=0.018). Mean OSDI values in Groups 3, 2, and 1 were 44.70, 26.53, and 5.18, respectively (p<0.001). Serum IgE was elevated in Group 2 and Group 3 patients compared to Group 1 (p< 0.001).

**Discussion:**

The diagnosis of VKC is primarily clinical, but immunological tests have been used to explore molecular and cellular mechanisms. Serum and tear inflammatory markers have been studied to identify biomarkers linked to immune responses and potential treatments. This study employed a flow cytometry-based cytokine bead array (CBA) to analyze inflammatory proteins in serum and tears. Overall, cytokine levels were higher in Group 2 than in Group 1, except for TNF-α and IL-10, and in Group 3 than in Group 2, except for IL-6. Significant positive correlations among tear parameters indicated the pro-inflammatory and immune-disturbed nature of VKC. Additionally, correlations between serum and tear parameters supported VKC as a Th2-mediated hypersensitivity. Higher mean OSDI scores (p < 0.001) in Group 3 showed that more severe VKC correlates with worse dry eye. The study also assessed quality of life, finding that 87.5% of patients sometimes rubbed their eyes, 52.5% used eye drops, and 57.5% experienced red eyes and burning sensations in the two weeks before evaluation. Limitations included a small sample size and the use of Schirmer strips for tear collection, which may dilute inflammatory mediators compared with basal tears collected by other methods.

**Conclusion:**

Tear and serum cytokines are largely associated with diseases severity in VKC patients. New treatment algorithms that involve systemic pathways may be developed.

## Introduction

Vernal keratoconjunctivitis (VKC) is a bilateral, chronic inflammatory eye disease associated with allergy and seasonal variation, with primary involvement of the bulbar and tarsal conjunctiva [1]. It is described as one of the most severe subtypes of allergic eye diseases [2], characterized by the predominant participation of mast cells, eosinophils, T-cells, macrophages, and basophils, with eosinophils being the most prominent cells causing various long-term sequelae. They are also found in tears, conjunctiva, and ulcers of patients with VKC, indicating their role in disease pathology [1]. The incidence can be as high as 30% in young children [3], and childhood disease is associated with an early presentation and increased rate of clinical morbidity.

Tear fluid consists of proteins, mucins, electrolytes, lipids, small organic molecules, and metabolites. Protein constituents in tears play a role in the defense mechanism of the ocular surface by protecting it from multiple pathogens and by modulating the wound-healing process [4]. The composition of tears reflects underlying tissue physiology, leading to increased interest in studying tear proteins and the relationship between physiological variation and protein composition. In recent years, tear analysis has gained increasing interest in ophthalmology because tear collection is relatively easy, non-invasive, painless, and convenient, and tears have been reported to have high protein content. Thus, research on tear fluid proteomics has been conducted in ocular diseases like dry eyes, keratoconus, glaucoma, diabetic retinopathy, pterygia, and conjunctivochalasis [5-12].

A literature search identified studies evaluating the role of tear proteomics in VKC. A correlation has been reported between tear cytokines, neuropeptides, and growth factors such as Nerve Growth Factor in active VKC. However, no study has established a diagnostic or prognostic biomarker for VKC. Furthermore, there is limited literature regarding research on tear inflammatory mediators in Indian patients with VKC, who predominantly exhibit a mixed form of the disease. Thus, the present study aimed to examine and understand the potential relationship between types of VKC and inflammatory mediators and, if possible, identify and validate a potential tear biomarker that can be utilized to diagnose and monitor the prognosis of VKC among young children of Indian origin, where the mixed type of VKC is more prevalent compared to the limbal or palpebral variety.

## Materials and methods

The prospective, non-randomized, observational study was conducted at the Cornea Services of a tertiary care ophthalmology centre, between July 2020 and September 2021. Approval from the Institutional Ethical Committee to conduct the study was obtained (INT/IEC/2022/000031). Fifty participants were included in the study, which were categorized into three groups:

Group 1: Control group comprised ten healthy participants without known allergies or undergoing any ocular surgery, aged between 8 and 16 years.

Group 2: Comprising 20 participants: patients of Moderate VKC.

Group 3: Consisting of 20 participants: patients of Severe VKC.

The classification proposed by Bonini et al. [13] was used to categorize VKC patients by disease severity as moderate or severe. The study group included patients of VKC who fulfilled the following inclusion/exclusion criteria:

Inclusion Criteria: 1) participants between 08-16 years old; 2) participants not on any oral/topical anti-inflammatory treatment (antihistamines/mast cell stabilizers/steroids) as well as immunomodulators including cyclosporine A and tacrolimus; 3) participants who had used any oral/topical medication but had stopped all oral and topical drugs at least 7 days before sampling were included in the study.

Exclusion criteria: 1) participants < 8 years or > 16 years of age; 2) participants with dry eyes (TBUT < 10 seconds and Schirmer’s < 10 mm), ocular surface disease index (OSDI) > 13, shield ulcer, previous history of ocular surgery/trauma, and steroid responders were excluded.

### Collection and Analysis of Sample

The participants were briefed about the study, and written consent was obtained. Demographic data of all included participants were collected. All eligible participants underwent a comprehensive ophthalmic examination, including calculation of the OSDI score. Corneal tomography using Oculus Pentacam was also performed for all patients and healthy controls. All participants underwent a complete analysis during a single visit.

### Serum collection

The serum investigations were performed as part of the standard treatment-of-care protocol for patients with moderate and severe VKC and were also conducted in ten healthy participants. 5 ml of blood was collected in a plain vial, and serum was separated and stored at -20 °C until further use.

### Tear collection

The more severely inflamed eye (decided on clinical examination) of VKC patients and the right eye of control group subjects were used for tear sampling. Tears were carefully collected using Schirmer’s strips in the precooled Eppendorf tubes, and the utmost care was taken to prevent any tear reflex. After collection, tears were stored at -80 °C until evaluated for analysis. Tears were eluted in 10 μl of PBS for processing by immersing the strip and agitating it, followed by centrifugation to separate the liquid containing the tear components from the paper matrix. Chilled PBS was added to the tear samples in Eppendorf tubes, and the tubes were incubated at 4 °C overnight. Following incubation, the eluent was collected by centrifugation and subsequently stored at -80 °C for subsequent use.

### Markers analyzed

(a) Th1/Th2/Th17 cytokines: IL-2-10,17, TNF-α, and IFN-γ were quantified in serum and tear samples using a bead-based multiplex assay, fluorescence-encoded beads, and flow cytometry (Becton Dickinson (BD) FACS LSR Fortessa cell analyzer).

(b) Metalloproteinases: TIMP-1 and 2 were quantified and determined in serum samples from VKC patients and controls using the ELISA method per the manufacturer’s instructions (Bioassay Technology Laboratory Shanghai, China).

(c) Total Serum IgE levels were also measured.

(d) Tear allergen assay and tear-specific IgE: tear allergens were assessed using Phadiatop for Inhalational antigens to detect the presence of respiratory antigens, and out of those positive for Phadiatop, the particular IgE was measured in tear sample using semi-quantitative-line-immunoassay-test strips coated with 21 different allergens (i.e. house dust mite mix, Dermatophagoides farina, Aspergillus fumigatus, Trichophyton mentagrophytes, Timothy grass, Cultivated rye, corn, Eucalyptus, Ragweed, Carnation flower, Sunflower, Cockroach, Cat, Dog, Pigeon feathers, Chicken feathers, Cotton, Straw dust, Jute, Sheep wool). The degree of positivity for different allergens was evaluated based on their concentrations, e.g., <0.35 Ku/l = Negative; 0.35-<3.5 Ku/L = low titer; 3.5-<100 Ku/L = high titer; >100 Ku/L = very high.

### Statistical Analysis

Data were coded, and SPSS v23 (IBM Corp) was used for data analysis. Descriptive statistics were elaborated as means + standard deviation, medians (IQRs) for continuous variables, and frequencies and percentages for categorical variables. Non-parametric Kruskal-Wallis Test/Wilcoxon test/Mann-Whitney test was used for data showing non-normal distribution. The chi-square test was used to compare categorical data in groups. Linear correlation amongst two continuous variables was explored using Spearman’s correlation for non-normally distributed data. p<0.05 was considered statistically significant. 

## Results

All participants in Group 3 were male, whereas 90% and 80% of participants in Groups 2 and 1 were male, respectively. The difference between the study and control groups in gender distribution was not statistically significant (*p* = 0.138). No significant difference was observed between groups concerning age (p = 0.955).

### Relationship between VKC Severity and control group

An overall increase in the levels of inflammatory mediators in the tears of subjects was observed in Group 2 compared to Group 1, except for TNF-α and IL-10, and in Group 3 as compared to Group 2, except for IL-6. Still, the differences were statistically non-significant (**Table 1-3**). In addition, no statistically significant differences were observed in serum cytokines or in TIMP-1 and TIMP-2 levels across groups (**Table 1-3**). The correlation between serum and tear markers in groups 1, 2, and 3 is described in **Table 4-7**.

**Table 1:** Relationship between different Groups regarding Tear and Serum Markers – Group 1

Sample	Parameters	Group 1			Kruskal-Wallis H	p value
		N	Mean ±SD	Median 50 (25-75)		
**Tear**	IL-17A (pg/mL)	6	9.48±7.643	8.00 (2.05-17.45)	.517	.772
	IFN-**ץ** (pg/mL)	10	1.53±1.424	0.83 (0.58-2.55)	.238	.888
	TNF-α (pg/mL)	6	4.48±4.487	2.55 (1.10-8.92)	5.405	.067
	IL-10 (pg/mL)	6	3.24±2.175	2.96 (1.17-5.54)	4.981	.083
	IL-6 (pg/mL)	10	55.85±83.253	17.06 (7.12-78.44)	.279	.870
	IL-4 (pg/mL)	7	1.98±1.685	2.23 (0.41-3.63)	2.290	.318
	IL-2 (pg/mL)	9	2.72±3.199	0.79 (0.26-5.38)	.137	.934
	Tear Allergen (PAU/L)	10	0.10±.000	0.10 (0.10-0.10)	3.350	.187
	Tear IgE for House Dust Mite (kU/L)	10	0.34±.000	0.34 (0.34-0.34)	2.586	.274
	Tear IgE for Dermatophagoides farinae (kU/L)	10	0.34±.000	0.34 (0.34-0.34)	3.580	.167
	Tear IgE for Sheep Wool (kU/L)	10	0.34±.000	0.34 (0.34-0.34)	.510	.775
**Serum**	IL-17A (pg/mL)	0	-	-	.129	.720
	IFN-**ץ** (pg/mL)	10	0.75±.242	0.71 (0.58-0.89)	3.618	.164
	TNF-α (p/mL)	3	0.61±.167	0.51 (0.51-.)	1.267	.531
	IL-10 (pg/mL)	9	2.05±2.045	1.18 (0.80-3.21)	4.917	.086
	IL-6 (pg/mL)	10	2.45±1.643	1.87 (1.12-3.67)	.198	.906
	IL-4 (pg/mL)	5	0.29±.153	0.27 (0.18-0.42)	2.509	.285
	IL-2 (pg/mL)	9	0.67±1.080	0.36 (0.15-0.53)	.604	.739
	TIMP1 (pg/mL)	10	665.65±1095.479	243.62 (210.12-656.68)	1.950	.377
	TIMP2 (pg/mL)	10	35.80 ±4.126	35.98 (31.64-39.09)	4.931	.085

**Table 2:** Relationship between different Groups regarding Tear and Serum Markers – Group 2

Sample	Parameters	Group2			Kruskal-Wallis H	p value
		N	Mean ±SD	Median 50 (25-75)		
**Tear**	IL-17A (pg/mL)	14	12.14±15.287	6.23 (4.58-15.42)	.517	.772
	IFN-**ץ** (pg/mL)	20	1.62±1.565	0.85 (.62-2.38)	.238	.888
	TNF-α (pg/mL)	16	1.64±1.626	1.15 (.53-2.36)	5.405	.067
	IL-10 (pg/mL)	17	2.57±3.318	1.13 (.45-3.31)	4.981	.083
	IL-6 (pg/mL)	20	70.55±123.456	20.13 (14.36-68.41)	.279	.870
	IL-4 (pg/mL)	17	3.14±5.102	0.41 (.30-3.79)	2.290	.318
	IL-2 (pg/mL)	20	2.96±4.684	0.96 (.61-3.66)	.137	.934
	Tear Allergen (PAU/L)	20	0.29±.582	0.10 (.10-.10)	3.350	.187
	Tear IgE for House Dust Mite (kU/L)	20	0.95±1.985	0.34 (.34-.34)	2.586	.274
	Tear IgE for Dermatophagoides farinae (kU/L)	20	0.85±1.849	0.34 (.34-.34)	3.580	.167
	Tear IgE for Sheep Wool (kU/L)	20	0.34±.020	0.34 (.34-.34)	.510	.775
**Serum**	IL-17A (pg/mL)	6	21.05± 44.585	3.88 (0.57-32.06)	.129	.720
	IFN-**ץ** (pg/mL)	18	1.50± 3.771	0.64 (0.42-0.89)	3.618	.164
	TNF-α (p/mL)	3	.40± .238	0.36 (0.19-.)	1.267	.531
	IL-10 (pg/mL)	18	.75±.552	0.81 (.21-1.03)	4.917	.086
	IL-6 (pg/mL)	20	4.62± 9.981	1.87 (.97-3.39)	.198	.906
	IL-4 (pg/mL)	9	.36±.228	0.32 (0.22-0.44)	2.509	.285
	IL-2 (pg/mL)	17	.46± .295	0.36 (0.26-0.60)	.604	.739
	TIMP1 (pg/mL)	20	292.34±115.944	262.48 (213.42-309.73)	1.950	.377
	TIMP2 (pg/mL)	20	36.07± 2.366	36.04 (33.94-38.22)	4.931	.085

**Table 3:** Relationship between different Groups regarding Tear and Serum Markers – Group 3

Sample	Parameters	Group3			Kruskal-Wallis H	p value
		N	Mean ±SD	Median 50 (25-75)		
**Tear**	IL-17A (pg/mL)	13	23.49±32.612	10.07 (2.98-25.21)	.517	.772
	IFN-**ץ** (pg/mL)	20	4.49±7.783	0.87(0.61-5.18)	.238	.888
	TNF-α (pg/mL)	11	4.90±4.319	3.36 (1.29-10.59)	5.405	.067
	IL-10 (pg/mL)	11	13.84±18.445	5.42 (2.15-19.16)	4.981	.083
	IL-6 (pg/mL)	20	56.48±99.619	31.55 (13.24-49.01)	.279	.870
	IL-4 (pg/mL)	14	8.26±13.948	3.13 (0.43-11.66)	2.290	.318
	IL-2 (pg/mL)	20	7.76±14.854	.96 (0.18-10.43)	.137	.934
	Tear Allergen (PAU/L)	20	0.76±1.325	0.10 (0.10-0.65)	3.350	.187
	Tear IgE for House Dust Mite (kU/L)	20	1.56±2.593	0.34 (0.34-0.34)	2.586	.274
	Tear IgE for Dermatophagoides farinae (kU/L)	20	0.85±.911	0.34 (0.34-1.88)	3.580	.167
	Tear IgE for Sheep Wool (kU/L)	20	0.34±.020	0.34 (0.34-0.34)	.510	.775
**Serum**	IL-17A (pg/mL)	7	15.99±36.017	2.16 (1.70-4.09)	.129	.720
	IFN-**ץ** (pg/mL)	20	1.30±3.452	0.45 (0.31-0.69)	3.618	.164
	TNF-α (p/mL)	6	0.66±.379	0.59 (0.28-1.09)	1.267	.531
	IL-10 (pg/mL)	14	1.11±.975	0.75(0.34-1.48)	4.917	.086
	IL-6 (pg/mL)	16	5.06±10.744	2.28 (1.01-3.48)	.198	.906
	IL-4 (pg/mL)	7	0.58±.398	0.41 (0.27-1.09)	2.509	.285
	IL-2 (pg/mL)	19	0.49±.683	0.36 (0.17-0.50)	.604	.739
	TIMP1 (pg/mL)	20	695.26± 953.825	293.44 (233.82-499.86)	1.950	.377
	TIMP2 (pg/mL)	20	33.99±2.368	34.51 (32.60-35.66)	4.931	.085

**Table 4:** Correlation between tear and serum parameters in Control and study groups (IL-17A rho, IFN- ץ rho, TNF-⍺ rho, 10 rho, IL-6 rho)

Groups	Parameters	Tear: IL-17A rho (p-value)	Tear: IFN-ץ rho (p-value)	Tear: TNF-⍺ rho (p-value)	Tear: IL-10 rho (p-value)	Tear: IL-6 rho (p-value)
**Group 1**	Serum: IL-17A	.	.	.	.	.
	Serum: IFN-ץ	.058 (.913)	-.711^*^(.021)	.200 (.704)	-.657 (.156)	-.413 (.235)
	Serum: TNF-⍺	.	-.866 (.333)	.	.	-.866 (.333)
	Serum: IL-10	-.100 (.873)	-.100 (.798)	.200 (.747)	.300 (.624)	-.350 (.356)
	Serum: IL-6	.086 (.872)	-.347 (.327)	-.580 (.228)	.377 (.461)	-.438 (.206)
	Serum: IL-4	-.632 (.368)	.410 (.493)	-1.000^**^ (.)	.000 (1.000)	-.051 (.935)
	Serum: IL-2	.900^*^ (.037)	.084 (.831)	-.200 (.747)	.500 (.391)	-.209 (.589)
	Serum: TIMP-1	-.086(.872)	.406 (.244)	-.771 (.072)	.029 (.957)	-.152 (.676)
	Serum: TIMP-2	-.771 (.072)	-.733^*^(.016)	.371(.468)	-.600 (.208)	-.152 (.676)
	Serum IgE	.	-.522 (.122)	.	.	.174 (.631)
**Group 2**	Serum: IL-17A	-.051 (.935)	.058 (.913)	.696 (.125)	.174 (.742)	.522 (.288)
	Serum: IFN-ץ	.128 (.676)	.269 (.281)	-.108 (.713)	.099 (.727)	.244 (.329)
	Serum: TNF-⍺	-.500 (.667)	-.500 (.667)	-.500 (.667)	.500 (.667)	-.500 (.667)
	Serum: IL-10	-.349 (.266)	-.230 (.359)	.073 (.805)	-.264 (.341)	.415 (.087)
	Serum: IL-6	-.164 (.574)	-.091 (.702)	-.128 (.636)	-.286 (.265)	-.438 (.053)
	Serum: IL-4	-.464 (.354)	-.178 (.647)	.155 (.741)	-.084 (.844)	-.286 (.456)
	Serum: IL-2	-.583 (.060)	-.316 (.217)	-.039 (.900)	-.323 (.261)	-.518^*^(.033)
	Serum: TIMP-1	.000(1.000)	.081 (.736)	-.490 (.054)	-.237 (.360)	-.400 (.081)
	Serum: TIMP-2	-.040 (.893)	-.192 (.417)	.459 (.073)	.101 (.701)	.006 (.980)
	Serum IgE	-.241 (.406)	-.182 (.442)	-.502^*^(.048)	-.189 (.468)	.155 (.515)
**Group 3**	Serum: IL-17A	.205 (.741)	-.126 (.788)	.500 (.391)	.300 (.624)	.090 (.848)
	Serum: IFN-ץ	.004 (.989)	-.218 (.355)	.594 (.054)	.297 (.375)	-.052 (.828)
	Serum: TNF-⍺	.800 (.200)	.812 (.050)	-.500 (.667)	-.500 (.667)	.464 (.354)
	Serum: IL-10	.269 (.484)	.104 (.724)	.536 (.215)	.234 (.613)	.220 (.449)
	Serum: IL-6	.103 (.777)	.049 (.858)	.333 (.381)	-.059 (.881)	.180 (.506)
	Serum: IL-4	.500 (.667)	.607 (.148)	.500 (.667)	.500 (.667)	.750 (.052)
	Serum: IL-2	-.059 (.856)	-.356 (.135)	-.202 (.575)	-.340 (.336)	-.104 (.671)
	Serum: TIMP-1	.234 (.442)	.003 (.990)	.492 (.124)	.273 (.416)	.359 (.120)
	Serum: TIMP-2	-.041 (.894)	-.095 (.690)	-.232 (.492)	-.228 (.501)	-.035 (.882)
	Serum IgE	-.514 (.073)	.463* (.040)	-.374 (.258)	-.523 (.099)	.462* (.040)

**Table 5:** Correlation between tear and serum parameters in Control and study groups (IL-4 rho, IL-2 rho, Allergen rho, IgE for House Dust Mite rho, IgE for Dermatophagoides farina rho, IgE for Sheep Wool rho)

Groups	Parameters	Tear: IL-4 rho (p-value)	Tear: IL-2 rho (p-value)	Tears Allergen rho (p-value)	Tear IgE for House Dust Mite rho (p-value)	Tear IgE for Dermatophagoides farina rho (p-value)	Tear IgE for Sheep Wool rho (p-value)
**Group 1**	Serum: IL-17A	.	.	.	.	.	.
	Serum: IFN-ץ	-.179 (.702)	-.644 (.061)	.	.	.	.
	Serum: TNF-⍺	.	-.866 (.333)	.	.	.	.
	Serum: IL-10	-.029 (.957)	-.667 (.071)	.	.	.	.
	Serum: IL-6	-.126 (.788)	-.477 (.194)	.	.	.	.
	Serum: IL-4	.000 (1.000)	-.211 (.789)	.	.	.	.
	Serum: IL-2	.086 (.872)	.048 (.911)	.	.	.	.
	Serum: TIMP-1	-.286 (.535)	.450 (.224)	.	.	.	.
	Serum: TIMP-2	-.321 (.482)	-.717^*^(.030)	.	.	.	.
	Serum IgE	.	.	.	.	.	.
**Group 2**	Serum: IL-17A	-.100 (.873)	.191 (.717)	-.133 (.802)	-.133 (.802)	-.133 (.802)	.
	Serum: IFN-ץ	.028 (.921)	.210 (.402)	.009 (.973)	.304 (.219)	.304 (.219)	-.328 (.184)
	Serum: TNF-⍺	-.500 (.667)	-.500 (.667)	1.000^**^ (.)	.866 (.333)	.866 (.333)	.000 (1.000)
	Serum: IL-10	-.237 (.377)	-.251 (.316)	.244 (.329)	.182 (.470)	.182 (.470)	.188 (.456)
	Serum: IL-6	-.604^*^(.010)	-.164 (.490)	-.098 (.681)	-.046 (.847)	-.046 (.847)	-.159 (.502)
	Serum: IL-4	-.164 (.726)	.092 (.813)	.391 (.298)	.414 (.268)	.414 (.268)	.069 (.860)
	Serum: IL-2	-.646^*^(.013)	-.391 (.121)	-.229 (.377)	-.257 (.319)	-.257 (.319)	.
	Serum: TIMP-1	-.325 (.204)	-.233 (.323)	.164 (.488)	.039 (.870)	.039 (.870)	.179 (.450)
	Serum: TIMP-2	.388 (.123)	.049 (.838)	.235 (.319)	.192 (.418)	.192 (.418)	.179 (.450)
	Serum IgE	-.172 (.510)	-.173 (.466)	.307 (.188)	.244 (.299)	.244 (.299)	.168 (.478)
**Group 3**	Serum: IL-17A	.086 (.872)	-.018 (.969)	.292 (.525)	.412 (.358)	.239 (.605)	-.103 (.826)
	Serum: IFN-ץ	.198 (.496)	-.245 (.298)	.024 (.921)	.047 (.844)	.050 (.833)	-.020 (.933)
	Serum: TNF-⍺	.400 (.600)	.779 (.068)	-.394 (.439)	-.531 (.278)	-.315 (.543)	.133 (.802)
	Serum: IL-10	.622 (.074)	.289 (.316)	-.276 (.340)	-.276 (.340)	-.276 (.340)	.
	Serum: IL-6	.382 (.221)	.074 (.786)	.015 (.955)	-.102 (.708)	.031 (.908)	.252 (.346)
	Serum: IL-4	.600 (.400)	.541 (.210)	.045 (.924)	.045 (.924)	.000 (1.000)	.
	Serum: IL-2	-.261 (.388)	-.270 (.264)	.216 (.375)	.222 (.362)	.214 (.379)	.
	Serum: TIMP-1	.191 (.513)	.118 (.622)	-.073 (.759)	-.027 (.910)	-.090 (.706)	-.099 (.677)
	Serum: TIMP-2	-.371 (.191)	-.254 (.280)	.320 (.169)	.331 (.154)	.280 (.231)	.020 (.934)
	Serum IgE	-.456 (.101)	-.464* (.040)	.190 (.423)	.166 (.486)	.192 (.416)	.076 (.749)

**Table 6:** Correlation between tear parameters in the Control and study groups (IL-17A rho, IFN-γ rho, TNF-α rho, IL-10 rho, IL-6 rho)

Groups	Parameters	Tear: IL-17A rho (p-value)	Tear: IFN-ץ rho (p-value)	Tear: TNF-⍺ rho (p-value)	Tear: IL-10 rho (p-value)	Tear: IL-6 rho (p-value)
**Group 1**	Tear IL-17A	1.000 (.)	.714 (.111)	.200 (.800)	.800 (.104)	.314 (.544)
	Tear IFN-**ץ**	.714 (.111)	1.000 (.)	-.371 (.468)	.943^**^(.005)	.164 (.651)
	Tear TNF-α	.200 (.800)	-.371 (.468)	1.000 (.)	-.300 (.624)	.771 (.072)
	Tear IL-10	.800 (.104)	.943^**^(.005)	-.300 (.624)	1.000 (.)	.486 (.329)
	Tear IL-6	.314 (.544)	.164 (.651)	.771 (.072)	.486 (.329)	1.000 (.)
	Tear IL-4	.800 (.104)	.143 (.760)	.543 (.266)	.429 (.397)	.714 (.071)
	Tear IL-2	.600 (.208)	.800^**^(.010)	-.429 (.397)	.943^**^(.005)	.700^*^(.036)
**Group 2**	Tear IL-17A	1.000 (.)	.773^**^ (.001)	.008 (.979)	.735^**^ (.004)	-.289 (.317)
	Tear IFN-**ץ**	.773^**^ (.001)	1.000 (.)	-.004 (.987)	.654^**^ (.004)	-.035 (.885)
	Tear TNF-α	.008 (.979)	-.004 (.987)	1.000 (.)	.028 (.922)	.443 (.085)
	Tear IL-10	.735^**^ (.004)	.654^**^ (.004)	.028 (.922)	1.000 (.)	.070 (.790)
	Tear IL-6	-.289 (.317)	-.035 (.885)	.443 (.085)	.070 (.790)	1.000 (.)
	Tear IL-4	.821^**^(.001)	.573^*^ (.016)	.325 (.257)	.826^**^(<.001)	.435 (.081)
	Tear IL-2	.676^**^(.008)	.787^**^(<.001)	.068 (.803)	.894^**^(<.001)	.150 (.528)
	Tear Allergen	-.072 (.806)	-.170 (.475)	-.099 (.714)	.186 (.474)	.174 (.463)
	Tear IgE for House Dust Mite	.207 (.477)	-.192 (.417)	-.173 (.522)	.294 (.253)	.079 (.739)
	Tear IgE for Dermatophagoides farinae	.207 (.477)	-.192 (.417)	-.173 (.522)	.294 (.253)	.079 (.739)
	Tear IgE for Sheep Wool	-.345 (.227)	-.060 (.802)	.140 (.604)	-.153 (.557)	.259 (.271)
**Group 3**	Tear IL-17A	1.000 (.)	.911^**^(<.001)	.117 (.765)	.867^**^ (.002)	.754^**^ (.003)
	Tear IFN-**ץ**	.911^**^(<.001)	1.000 (.)	.437 (.179)	.966^**^(<.001)	.664^**^ (.001)
	Tear TNF-α	.117 (.765)	.437 (.179)	1.000 (.)	.564 (.071)	.524 (.098)
	Tear IL-10	.867^**^ (.002)	.966^**^(<.001)	.564 (.071)	1.000 (.)	.401 (.222)
	Tear IL-6	.754^**^ (.003)	.664^**^ (.001)	.524 (.098)	.401 (.222)	1.000 (.)
	Tear IL-4	.818^**^ (.002)	.906^**^(<.001)	.729^*^ (.017)	.891^**^ (.001)	.710^**^(.004)
	Tear IL-2	.923^**^(<.001)	.830^**^(<.001)	.437 (.179)	.980^**^(<.001)	.577^**^ (.008)
	Tear Allergen	.122 (.690)	-.185 (.435)	-.324 (.330)	.081 (.813)	-.251 (.286)
	Tear IgE for House Dust Mite	.155 (.614)	-.280 (.232)	-.150 (.659)	.100 (.769)	-.156 (.511)
	Tear IgE for Dermatophagoides farinae	.114 (.710)	-.181 (.446)	-.336 (.312)	.075 (.827)	-.290 (.214)
	Tear IgE for Sheep Wool	.000 (1.000)	.179 (.449)	-.301 (.369)	.000 (1.000)	-.259 (.271)

**Table 7:** Correlation between tear parameters in the Control and study groups (IL-4 rho, IL-2 rho, Allergen rho, IgE for House Dust Mite rho, IgE for Dermatophagoides farina rho, IgE for Sheep Wool rho)

Groups	Parameters	Tear: IL-4 rho (p-value)	Tear: IL-2 rho (p-value)	Tears: Allergen rho (p-value)	Tear IgE for House Dust Mite rho (p-value)	Tear IgE for Dermatophagoides farina rho (p-value)	Tear IgE for Sheep Wool rho (p-value)
**Group 1**	Tear IL-17A	.800 (.104)	.600 (.208)	.	.	.	.
	Tear IFN-** ץ**	.143 (.760)	.800^**^(.010)	.	.	.	.
	Tear TNF-α	.543 (.266)	-.429 (.397)	.	.	.	.
	Tear IL-10	.429 (.397)	.943^**^(.005)	.	.	.	.
	Tear IL-6	.714 (.071)	.700^*^ (.036)	.	.	.	.
	Tear IL-4	1.000 (.)	.464 (.294)	.	.	.	.
	Tear IL-2	.464 (.294)	1.000 (.)	.	.	.	.
**Group 2**	Tear IL-17A	.821^**^(.001)	.676^**^(.008)	-.072 (.806)	.207 (.477)	.207 (.477)	-.345 (.227)
	Tear IFN-** ץ**	.573^*^ (.016)	.787^**^(<.001)	-.170 (.475)	-.192 (.417)	-.192 (.417)	-.060 (.802)
	Tear TNF-α	.325 (.257)	.068 (.803)	-.099 (.714)	-.173 (.522)	-.173 (.522)	.140 (.604)
	Tear IL-10	.826^**^(<.001)	.894^**^(<.001)	.186 (.474)	.294 (.253)	.294 (.253)	-.153 (.557)
	Tear IL-6	.435 (.081)	.150 (.528)	.174 (.463)	.079 (.739)	.079 (.739)	.259 (.271)
	Tear IL-4	1.000 (.)	.779^**^(<.001)	.164 (.528)	.233 (.369)	.233 (.369)	-.051 (.845)
	Tear IL-2	.779^**^(<.001)	1.000	.078 (.745)	.172 (.468)	.172 (.468)	-.140 (.557)
	Tear Allergen	.164 (.528)	.078 (.745)	1.000 (.)	.833^**^(<.001)	.833^**^ (<.001)	.480^*^(.032)
	Tear IgE for House Dust Mite	.233 (.369)	.172 (.468)	.833^**^(<.001)	1.000^**^ (.)	1.000^**^ (.)	-.076 (.749)
	Tear IgE for Dermatophagoides farinae	.233 (.369)	.172 (.468)	.833^**^(<.001)	1.000^**^ (.)	1.000 (.)	-.076 (.749)
	Tear IgE for Sheep Wool	-.051 (.845)	-.140 (.557)	.480^*^ (.032)	-.076 (.749)	-.076 (.749)	1.000
**Group 3**	Tear IL-17A	.818^**^ (.002)	.923^**^(<.001)	.122 (.690)	.155 (.614)	.114 (.710)	.000 (1.000)
	Tear IFN-** ץ**	.906^**^(<.001)	.830^**^(<.001)	-.185 (.435)	-.280 (.232)	-.181 (.446)	.179 (.449)
	Tear TNF-α	.729^*^ (.017)	.437 (.179)	-.324 (.330)	-.150 (.659)	-.336 (.312)	-.301 (.369)
	Tear IL-10	.891^**^ (.001)	.980^**^(<.001)	.081 (.813)	.100 (.769)	.075 (.827)	.000 (1.000)
	Tear IL-6	.710^**^ (.004)	.577^**^(.008)	-.251 (.286)	-.156 (.511)	-.290 (.214)	-.259 (.271)
	Tear IL-4	1.000 (.)	.921^**^(<.001)	-.396 (.161)	-.420 (.135)	-.392 (.165)	-.034 (.907)
	Tear IL-2	.921^**^(<.001)	1.000 (.)	-.151 (.526)	-.245 (.298)	-.140 (.555)	.179 (.449)
	Tear Allergen	-.396 (.161)	-.151 (.526)	1.000 (.)	.918^**^(<.001)	.987^**^ (<.001)	.288 (.219)
	Tear IgE for House Dust Mite	-.420 (.135)	-.245 (.298) .	.918^**^(<.001)	1.000 (.)	.860^**^ (<.001)	-.114 (.633)
	Tear IgE for Dermatophagoides farinae	-.392 (.165)	-.140 (.555)	.987^**^(<.001)	.860^**^(<.001)	1.000 (.)	.397 (.083)
	Tear IgE for Sheep Wool	-.034 (.907)	.179 (.449)	.288 (.219)	-.114 (.633)	.397 (.083)	1.000 (.)

*Tear Allergen, Tear IgE for House Dust Mite, Dermatophagoides farina, and Sheep Wool were not detectable in tears of Group 1 patients*

### Relationship with Keratoconus

Keratoconus was absent in 85% and 50% of subjects in groups 2 and 3, respectively. A statistically significant difference was observed between the groups regarding the presence of keratoconus (*p*= 0.018). All the studied tear markers in groups 2 and 3 showed a non-significant difference with respect to the presence or absence of keratoconus, except for tear-specific IgE for sheep wool (p = 0.017) in group 2, where the difference was significant (**Table 8, 9**). All the studied serum parameters showed no statistically significant difference regarding the presence or absence of keratoconus in both groups 2 and 3.

**Table 8:** Relationship between Tear parameters and Keratoconus in groups 2 and 3 - Keratoconus (Absent)

Groups	Parameters	Keratoconus (Absent)			Mann-Whitney U	Wilcoxon W	p-value
		N	Mean ± SD	Median 50 (25-75)			
**Group 2**	IL-17A (pg/mL)	11	13.33±16.969	6.38 (5.11-15.94)	11.500	17.500	.435
	IFN-ץ (pg/mL)	17	1.60±1.605	0.89 (0.58-2.25)	20.000	173.000	.560
	TNF-α (pg/mL)	14	1.50±1.657	0.91 (0.49-1.96)	5.000	110.000	.152
	IL-10 (pg/mL)	14	2.61±3.511	1.62 (0.44-3.28)	19.500	124.500	.850
	IL-6 (pg/mL)	17	69.48±133.186	18.77 (10.40-34.23)	11.000	164.000	.125
	IL-4 (pg/mL)	14	2.92±5.014	0.76 (0.26-3.76)	18.000	123.000	.705
	IL-2 (pg/mL)	17	2.96±4.949	0.93 (0.60-3.61)	20.000	173.000	.560
	Tears Allergen (PAU/L)	17	0.29±.619	0.10 (0.10-0.10)	21.000	174.000	.444
	Tear IgE for House Dust Mite (kU/L)	17	1.06±2.144	0.34 (0.34-0.34)	22.500	28.500	.542
	Tear IgE for Dermatophagoides Farinae (kU/L)	17	0.94±2.001	0.34 (0.34-0.34)	22.500	28.500	.542
	Tear IgE for Sheep Wool (kU/L)	17	0.34±.000	0.34 (0.34-0.34)	17.000	170.000	.017 (S)
**Group 3**	IL-17A (pg/mL)	6	25.64±34.380	12.89 (5.98-42.57)	17.000	45.000	.628
	IFN-ץ (pg/mL)	10	4.47±8.081	1.58 (0.64-4.51)	39.500	94.500	.436
	TNF-α (pg/mL)	7	3.70±4.068	2.23 (0.23-8.07)	5.500	33.500	.109
	IL-10 (pg/mL)	7	10.53±17.926	4.45 (0.21-10.96)	8.000	36.000	.315
	IL-6 (pg/mL)	10	45.56±36.505	36.12 (16.79-64.81)	36.000	91.000	.315
	IL-4 (pg/mL)	8	9.56±18.015	2.01 (0.50-11.97)	21.000	57.000	.755
	IL-2 (pg/mL)	10	9.18±18.949	1.72 (0.28-9.45)	41.500	96.500	.529
	Tears Allergen (PAU/L)	10	0.62±1.407	0.10 (0.10-0.29)	45.000	100.000	.739
	Tear IgE for House Dust Mite (kU/L)	10	1.15±2.568	0.34 (0.34-0.34)	41.500	96.500	.529
	Tear IgE for Dermatophagoides Farinae (kU/L)	10	0.75±.864	0.34 (0.34-0.85)	45.000	100.000	.739
	Tear IgE for Sheep Wool (kU/L)	10	0.35±.028	0.34 (0.34-0.34)	45.000	100.000	.739

**Table 9:** Relationship between Tear parameters and Keratoconus in groups 2 and 3 - Keratoconus (Present)

Groups	Parameters	Keratoconus (Present)			Mann-Whitney U	Wilcoxon W	p-value
		N	Mean ± SD	Median 50(25-75)			
**Group 2**	IL-17A (pg/mL)	3	7.78±6.556	5.11 (2.98 -.)	11.500	17.500	.435
	IFN-ץ (pg/mL)	3	1.75±1.622	0.81 (0.81-.)	20.000	173.000	.560
	TNF-α (pg/mL)	2	2.63±1.322	2.63 (1.69-.)	5.000	110.000	.152
	IL-10 (pg/mL)	3	2.37±2.807	0.83 (0.67-.)	19.500	124.500	.850
	IL-6 (pg/mL)	3	76.61±53.120	76.49 (23.55-.)	11.000	164.000	.125
	IL-4 (pg/mL)	3	4.17±6.553	0.41 (0.37- .)	18.000	123.000	.705
	IL-2 (pg/mL)	3	2.92±3.533	1.13 (0.64 -.)	20.000	173.000	.560
	Tears Allergen (PAU/L)	3	0.32±.381	0.10 (0.10- .)	21.000	174.000	.444
	Tear IgE for House Dust Mite (kU/L)	3	0.34±.000	0.34 (0.34-0.34)	22.500	28.500	.542
	Tear IgE for Dermatophagoides Farinae (kU/L)	3	0.34±.000	0.34 (0.34-0.34)	22.500	28.500	.542
	Tear IgE for Sheep Wool (kU/L)	3	0.37±.000	0.34 (0.34-.)	17.000	170.000	.017(S)
**Group 3**	IL-17A (pg/mL)	7	21.65±33.669	4.44 (2.98-24.93)	17.000	45.000	.628
	IFN-ץ (pg/mL)	10	4.51±7.911	0.77 (0.51-6.71)	39.500	94.500	.436
	TNF-α (pg/mL)	4	6.99±4.461	7.41 (2.71-10.86)	5.500	33.500	.109
	IL-10 (pg/mL)	4	19.64±20.517	13.22 (4.27-41.32)	8.000	36.000	.315
	IL-6 (pg/mL)	10	67.40±139.114	17.85 (6.97-50.90)	36.000	91.000	.315
	IL-4 (pg/mL)	6	6.54±6.727	5.12 (0.38-12.42)	21.000	57.000	.755
	IL-2 (pg/mL)	10	6.35±10.113	0.60 (.16-14.47)	41.500	96.500	.529
	Tears Allergen (PAU/L)	10	0.89±1.298	0.10 (0.10-2.39)	45.000	100.000	.739
	Tear IgE for House Dust Mite (kU/L)	10	1.97±2.689	0.34 (0.34-4.91)	41.500	96.500	.529
	Tear IgE for Dermatophagoides Farinae (kU/L)	10	0.96±.990	0.34 (0.34-2.39)	45.000	100.000	.739
	Tear IgE for Sheep Wool (kU/L)	10	0.34±.000	0.34 (0.34-0.34)	45.000	100.000	.739

### Relationship with OSDI

Mean OSDI values among participants of Groups 3, 2 and 1 were 44.70, 26.53 and 5.18, respectively. A statistically significant difference was observed between the three groups regarding OSDI value (p<0.001). OSDI was further sub-grouped into moderate (Index between 13-23) and severe (Index > 33). In both groups 2 and 3, no significant difference was observed in levels of tear cytokines, tear allergens, and tear-specific IgE regarding the severity of OSDI (**Tables 10,11**). The difference in levels of serum cytokines, TIMP-1 and TIMP-2, between moderate and severe OSDI in groups 2 and 3 was statistically non-significant.

**Table 10:** Relationship between Tear parameters and OSDI Moderate

Groups	Parameters	OSDI Moderate			Mann-Whitney U	Wilcoxon W	p-value
		N	Mean ± SD	Median 50 (25-75)			
**Group 2**	IL-17A (pg/mL)	05	9.24+7.333	6.07 (3.15-16.91)	14.50	35.50	.927
	IFN-ץ (pg/mL)	07	1.54+1.304	0.89 (0.56-3.21)	20.00	48.00	.886
	TNF-α (pg/mL)	05	2.24+2.664	0.59 (0.36-4.95)	15.00	36.00	1.000
	IL-10 (pg/mL)	06	2.32+2.559	0.93 (0.44-0.93)	12.00	33.00	.583
	IL-6 (pg/mL)	07	95.66+185.698	20.53 (13.26-76.49)	19.00	40.00	.775
	IL-4 (pg/mL)	06	3.33+4.941	0.39 (0.22-8.25)	13.50	34.50	.784
	IL-2 (pg/mL)	07	2.78+3.690	0.93 (0.60-6.99)	18.00	46.00	.667
	Tears Allergen (PAU/L)	07	0.10+0.00	0.10 (0.10-0.10)	14.00	42.00	.112
	Tear IgE for House Dust Mite (kU/L)	07	0.34+0.00	0.34 (0.34-0.34)	17.50	45.50	.280
	Tear IgE for Dermatophagoides Farinae (kU/L)	07	0.34+0.00	0.34 (0.34-0.34)	17.50	45.50	.280
	Tear IgE for Sheep Wool (kU/L)	07	0.34+0.00	0.34 (0.34-0.34)	17.50	45.50	.280
**Group 3**	IL-17A (pg/mL)	02	59.36+48.691	59.36 (24.93-.)	3.00	69.00	.114
	IFN-ץ (pg/mL)	04	9.70+12.312	5.61 (0.73-22.75)	17.00	153.00	.156
	TNF-α (pg/mL)	03	5.66+4.379	4.15 (2.23-.)	9.50	45.50	.609
	IL-10 (pg/mL)	03	23.22+25.291	19.16 (0.21-.)	8.50	44.50	.474
	IL-6 (pg/mL)	04	81.71+36.536	89.57 (43.67-111.90)	8.00	144.00	.023
	IL-4 (pg/mL)	04	16.17+24.694	5.90 (0.54-42.06)	16.00	71.00	.572
	IL-2 (pg/mL)	04	21.78+28.899	12.68 (0.32-52.35)	19.50	155.50	.236
	Tears Allergen (PAU/L)	04	1.22+2.235	0.10 (0.10-3.45)	30.00	166.00	.804
	Tear IgE for House Dust Mite (kU/L)	04	2.37+4.060	0.34 (0.34-6.43)	28.50	164.50	.636
	Tear IgE for Dermatophagoides Farinae (kU/L)	04	0.85+1.025	0.34 (0.34-1.88)	32.00	168.00	1.000
	Tear IgE for Sheep Wool (kU/L)	04	0.34+0.00	0.34 (0.34-0.34)	30.00	40.00	.617

**Table 11:** Relationship between Tear parameters and OSDI Severe

Groups	Parameters	OSDI Severe			Mann-Whitney U	Wilcoxon W	p-value
		N	Mean ± SD	Median 50(25-75)			
**Group 2**	IL-17A (pg/mL)	06	8.46±5.855	6.79 (2.98-15.42)			
	IFN-ץ (pg/mL)	06	1.51±0.898	1.29 (0.78-2.24)	14.50	35.50	.927
	TNF-α (pg/mL)	06	1.12±0.582	1.19 (0.47-1.71)	20.00	48.00	.886
	IL-10 (pg/mL)	05	2.17±1.226	2.44 (0.90-3.31)	15.00	36.00	1.000
	IL-6 (pg/mL)	06	36.03±46.218	19.26 (15.14-49.47)	12.00	33.00	.583
	IL-4 (pg/mL)	05	1.24±0.969	1.10 (0.32-2.23)	19.00	40.00	.775
	IL-2 (pg/mL)	06	2.06±1.486	1.92 (0.64-3.56)	13.50	34.50	.784
	Tears Allergen (PAU/L)	06	0.63±0.998	0.10 (0.10-1.22)	18.00	46.00	.667
	Tear IgE for House Dust Mite (kU/L)	06	1.01±1.649	0.34 (0.34-1.35)	14.00	42.00	.112
	Tear IgE for Dermatophagoides Farinae (kU/L)	06	0.68±0.837	0.34 (0.34-0.85)	17.50	45.50	.280
	Tear IgE for Sheep Wool (kU/L)	06	0.36±0.37	0.34 (0.34-0.36)	17.50	45.50	.280
**Group 3**	IL-17A (pg/mL)	11	16.97±27.112	7.57 (2.98-20.85)	17.50	45.50	.280
	IFN-ץ (pg/mL)	16	3.19±6.115	0.77 (0.54-3.66)	3.00	69.00	.114
	TNF-α (pg/mL)	08	4.62±4.565	2.80 (0.53-10.02)	17.00	153.00	.156
	IL-10 (pg/mL)	08	10.32±15.855	4.94 (2.41-10.09)	9.50	45.50	.609
	IL-6 (pg/mL)	16	50.17±109.959	19.74 (10.09-40.26)	8.50	44.50	.474
	IL-4 (pg/mL)	10	5.10±6.240	3.13 (0.41-7.75)	8.00	144.00	.023
	IL-2 (pg/mL)	16	4.26±6.849	0.96 (0.17-6.61)	16.00	71.00	.572
	Tears Allergen (PAU/L)	16	0.64±1.074	0.10 (0.10-0.65)	19.50	155.50	.236
	Tear IgE for House Dust Mite (kU/L)	16	1.36±2.237	0.34 (0.34-0.34)	30.00	166.00	.804
	Tear IgE for Dermatophagoides Farinae (kU/L)	16	0.85±0.917	0.34 (0.34-1.88)	28.50	164.50	.636
	Tear IgE for Sheep Wool (kU/L)	16	0.35±0.022	0.34 (0.34-0.34)	32.00	168.00	1.000

### Relationship with Serum IgE

The mean value of serum IgE in Groups 1, 2 and 3 was 119.88±163.36, 621.50±1212.67 and 1279.62±1669.92, respectively. A significant difference was found between Group 1 vs. Group 2 (p=0.020), Group 1 vs. Group 3 (p<.001), and Group 2 vs. Group 3 (p=0.017) in serum IgE. The serum IgE group was further divided into two subgroups: serum IgE < 250 and >250 kU/L. Most participants in group 2 (65%) and group 3 (90%) had serum IgE> 250 kU/L, whereas 90% of participants in group 1 had serum IgE < 250 kU/L, and this difference between groups was found to be statistically significant (*p*<0.001). All the studied tear markers in groups 1, 2, and 3 showed a non-significant difference concerning serum IgE levels except for tear IFN-γ, IL-6, and IL-2 in group 3. The difference was significant in some cases (**Table 12, 13**). However, no significant differences in serum parameters were found among Groups 1, 2, and 3 for serum IgE.

**Table 12:** Relationship between Tear parameters and Serum IgE (kU/L) < 250

Groups	Parameters	Serum IgE (kU/L) < 250			Mann-Whitney U	Wilcoxon W	p-value
		N	Mean ± SD	Median 50 (25-75)			
**Group 1**	IL-17A (pg/mL)	6	-	-	-	-	-
	IFN-ץ (pg/mL)	9	.000	1.000	.000	1.000	.117
	TNF-α (pg/mL)	6	-	-	-	-	-
	IL-10 (pg/mL)	6	-	-	-	-	-
	IL-6 (pg/mL)	9	3.000	48.000	3.000	48.000	.602
	IL-4 (pg/mL)	7	-	-	-	-	-
	IL-2 (pg/mL)	9	-	-	-	-	-
	Tears Allergen (PAU/L)	9	4.500	5.500	4.500	5.500	1.000
	Tear IgE for House Dust Mite (kU/L)	9	4.500	5.500	4.500	5.500	1.000
	Tear IgE for Dermatophagoides Farinae (kU/L)	9	4.500	5.500	4.500	5.500	1.000
	Tear IgE for Sheep Wool (kU/L)	9	4.500	5.500	4.500	5.500	1.000
**Group 2**	IL-17A (pg/mL)	5	16.000	61.000	16.000	61.000	.385
	IFN-ץ (pg/mL)	7	35.500	126.500	35.500	126.500	.427
	TNF**-**α (pg/mL)	4	8.000	86.000	8.000	86.000	.052
	IL-10 (pg/mL)	6	25.500	91.500	25.500	91.500	.450
	IL-6 (pg/mL)	7	37.000	65.000	37.000	65.000	.501
	IL-4 (pg/mL)	5	23.500	101.500	23.500	101.500	.492
	IL-2 (pg/mL)	7	36.000	127.000	36.000	127.000	.451
	Tears Allergen (PAU/L)	7	35.000	63.000	35.000	63.000	.181
	Tear IgE for House Dust Mite (kU/L)	7	38.500	66.500	38.500	66.500	.287
	Tear IgE for Dermatophagoides Farinae (kU/L)	7	38.500	66.500	38.500	66.500	.287
	Tear IgE for Sheep Wool (kU/L)	7	42.000	70.000	42.000	70.000	.463
**Group 3**	IL-17A (pg/mL)	13	2.000	68.000	2.000	68.000	.075
	IFN-ץ (pg/mL)	20	2.000	173.000	2.000	173.000	**.043 (S)**
	TNF-α (pg/mL)	11	4.000	49.000	4.000	49.000	.238
	IL-10 (pg/mL)	11	2.000	47.000	2.000	47.000	.098
	IL-6 (pg/mL)	20	2.000	173.000	2.000	173.000	**.044 (S)**
	IL-4 (pg/mL)	14	3.000	81.000	3.000	81.000	.100
	IL-2 (pg/mL)	20	2.000	173.000	2.000	173.000	**.043 (S)**
	Tears Allergen (PAU/L)	20	0.10±.000	0.10 (0.10-0.10-.)	13.000	16.000	.408
	Tear IgE for House Dust Mite (kU/L)	20	0.34±.000	0.34 (0.34-0.34)	14.000	17.000	.471
	Tear IgE for Dermatophagoides Farinae (kU/L)	20	0.34±.000	0.34 (0.34- 0.34)	13.000	16.000	.402

**Table 13:** Relationship between Tear parameters and Serum IgE (k/L) > 250

Groups	Parameters	Se IgE (k/L) > 250			Mann-Whitney U	Wilcoxon W	p-value
		N	Mean ± SD	Median 50(25-75)			
**Group 1**	IL-17A (pg/mL)	0	-	-	-	-	-
	IFN-ץ (pg/mL)	1	0.22±-	0.22 (0.22-0.22)	.000	1.000	.117
	TNF-α (pg/mL)	0	-	-	-	-	-
	IL-10 (pg/mL)	0	-	-	-	-	-
	IL-6 (pg/mL)	1	39.59±-	39.59 (39.59-39.59)	3.000	48.000	.602
	IL-4 (pg/mL)	0	-	-	-	-	-
	IL-2 (pg/mL)	0	-	-	-	-	-
	Tears Allergen (PAU/L)	1	0.10± -	0.10 (0.10-0.10)	4.500	5.500	1.000
	Tear IgE for House Dust Mite (kU/L)	1	0.34± -	0.34 (0.34-0.34)	4.500	5.500	1.000
	Tear IgE for Dermatophagoides Farinae (kU/L)	1	0.34± -	0.34 (0.34-0.34)	4.500	5.500	1.000
	Tear IgE for Sheep Wool (kU/L)	1	0.34± -	0.34 (0.34-0.34)	4.500	5.500	1.000
**Group 2**	IL-17A (pg/mL)	9	8.27±6.501	6.07 (2.98-15.60)	16.000	61.000	.385
	IFN-ץ (pg/mL)	13	1.30±.979	0.81 (0.58-2.13)	35.500	126.500	.427
	TNF**-**α (pg/mL)	12	1.43±1.768	0.63 (0.45-1.56)	8.000	86.000	.052
	IL-10 (pg/mL)	11	1.81±1.736	1.03 (.45-3.24)	25.500	91.500	.450
	IL-6 (pg/mL)	13	93.78±148.801	20.53 (15.77-147.01)	37.000	65.000	.501
	IL-4 (pg/mL)	12	1.69±2.181	0.41 (0.28-3.39)	23.500	101.500	.492
	IL-2 (pg/mL)	13	1.98±2.508	0.93 (0.60-3.24)	36.000	127.000	.451
	Tears Allergen (PAU/L)	13	0.40±.708	0.10 (0.10-0.43)	35.000	63.000	.181
	Tear IgE for House Dust Mite (kU/L)	13	1.28±2.430	0.34 (0.34-0.34)	38.500	66.500	.287
	Tear IgE for Dermatophagoides Farinae (kU/L)	13	1.12±2.276	0.34 (0.34-0.34)	38.500	66.500	.287
	Tear IgE for Sheep Wool (kU/L)	13	0.35±.025	0.34 (0.34-0.34)	42.000	70.000	.463
**Group 3**	IL-17A (pg/mL)	11	16.88±26.819	7.57 (2.98-20.85)	2.000	68.000	.075
	IFN-ץ (pg/mL)	18	3.03±6.212	0.77 (0.58-2.79)	2.000	173.000	**.043 (S)**
	TNF-α (pg/mL)	9	4.34±4.329	2.23 (0.79-9.33)	4.000	49.000	.238
	IL-10 (pg/mL)	9	9.38±15.729	4.45 (1.18-9.22)	2.000	47.000	.098
	IL-6 (pg/mL)	18	33.26±31.398	26.15 (12.04-41.71)	2.000	173.000	**.044 (S)**
	IL-4 (pg/mL)	12	7.27±14.834	2.01 (.36-5.25)	3.000	81.000	.100
	IL-2 (pg/mL)	18	5.91±14.504	0.60 (0.17-4.48)	2.000	173.000	**.043 (S)**
	Tears Allergen (PAU/L)	18	.83±1.380	0.10 (0.10-1.20)	13.000	16.000	.408
	Tear IgE for House Dust Mite (kU/L)	18	1.70±2.706	0.34 (0.34-1.35)	14.000	17.000	.471
	Tear IgE for Dermatophagoides Farinae (kU/L)	18	1.70±2.706	0.34 (0.34-1.35)	13.000	16.000	.402
	Tear IgE for Sheep Wool (kU/L)	18	0.91±.945	0.34 (0.34-2.39)	17.000	20.000	.739

## Discussion

The diagnosis of VKC is primarily clinical; however, various immunological tests have been performed to identify molecular or cellular mechanisms associated with the disease. Various serum and tear inflammatory markers have been studied to identify biomarkers that may help elucidate the immune mechanisms underlying potential therapeutic targets. Still, no single mediator has been identified that could be used for diagnostic or therapeutic purposes. Compared with serum, tear samples are easier to collect (via the Schirmer Strip or micropipette methods) and provide information on tear cytology and inflammatory mediators. Thus, the present study was conducted to correlate tear and serum inflammatory markers with disease severity in VKC.

This study used a cytokine bead array (CBA) based on flow cytometry to analyze serum and tear inflammatory proteins. This technique has been proven to have sensitivity equivalent to conventional ELISA [1]. It has several advantages over traditional ELISA. The required sample volume is just one-seventh of the quantity necessary for a conventional ELISA assay, as it detects seven analytes in a single sample, uses a single set of diluted standards to generate a standard curve for each analyte, and is less time-consuming than a single ELISA and the provision of results, which would typically require seven conventional ELISAs.

In the present study, the overall levels of all tear and serum cytokines were elevated in Group 2 compared to Group 1, except for TNF alpha and IL-10, and in Group 3 compared to Group 2, except for IL-6, which is in concordance with the previous studies [14,15-17]. However, the differences in marker levels between the groups were found to be statistically nonsignificant in the present study. In Groups 2 and 3, three and five patients, respectively, showed elevated levels of tear allergens on the Phadiatop assay. The current study evaluated tear-specific IgE to House dust mites (Dermatophagoides farinae) and Sheep Wool in patients with elevated tear allergen levels on the Phadiatop assay. Subjects with elevated tear allergens also had higher levels of tear-specific IgE. The detected levels of tear-specific IgE were minimal in the other patients in the study and in the control groups. According to Mimura et al. [18], their study demonstrated that tear fluid IgE might be useful in establishing a diagnosis of indoor allergen-related allergic conjunctivitis. Serum IL-10 levels were higher in healthy controls than in patients with active disease (p = 0.086). IL-10, an anti-inflammatory cytokine [14], decreases antigen-dependent cellular activation and thus prevents the clustering of eosinophils at various inflammatory sites. This may explain the increased serum levels observed in healthy controls in this study and further supports the role of reduced anti-inflammatory mediators in the pathogenesis of the disease. Serum IL-17A was detected only in patients with active disease in the current study, consistent with findings from Validad et al. [19] and Zicari et al. [20], which demonstrated that elevated serum IL-17A levels in patients with VKC support its role in VKC.

In the present study, significant positive correlations between different tear parameters were observed, demonstrating the pro-inflammatory nature of this disease and disturbed immune response in patients with VKC. In addition, significant correlations between certain serum and tear parameters support the conclusion that VKC is predominantly a Th2-mediated cellular hypersensitivity response.

Excessive and frequent eye rubbing is a documented factor common to both VKC and keratoconus, leading to increased levels of inflammatory mediators such as IL-6, MMP-13, and TNF-alpha in both tears and serum, thereby triggering the death of stromal keratocytes in patients with accentuated IL-6 sensitivity [20]. In the present study, no significant difference was observed in levels of studied tear and serum markers concerning the presence or absence of keratoconus in both group 2 and group 3, except for levels of tear-specific IgE for sheep wool (*p*=0.017) in group 2, which could be explained based on a smaller sample size of the study.

Dry eyes have been documented in patients with VKC because of modifications in tear film mucin, dropout in the meibomian gland, and reduced density of corneal nerves [3]. In their study, Akil et al. [21] showed a 12% prevalence of dry eye in patients with VKC. OSDI is an established criterion for classifying the type of dry eye, and it was evaluated in all the patients with active disease and healthy controls in the present study. Significantly higher mean OSDI values (*p* < 0.001) in Group 3 compared with Groups 2 and 1 indicate that higher clinical grades of VKC are typically associated with more severe dry eye. In patients with severe VKC, significantly higher tear IL-6 was observed in those with moderate OSDI than in those with severe OSDI (*p = 0.023*). This finding was somewhat inconsistent with expectations, as higher VKC grades are typically associated with more severe dry eye. A possible explanation is the significantly lower number of patients in the moderate OSDI (4) group compared with the severe OSDI (16) group, and the wide range of marker levels in that group. The present study was conducted during the ongoing COVID-19 pandemic, which led to increased screen time among all children due to online academic sessions and may further increase dry eyes and, hence, exacerbate disease in patients with active VKC.

Mean serum IgE (kU/L) was significantly raised in patients with both severe (1279.62±1669.923) and moderate (621.50±1212.672) VKC as compared to controls (119.88±163.363) (p < 0.001), which has also been reported in a previous study by Nebbioso et al. [22]. In group 3, patients with serum IgE < 250 kU/L showed significantly increased levels of tear IFN-, Tear IL-6, and Tear IL-2 in comparison to serum IgE > 250 kU/L (p = 0.043,0.044 and 0.043, respectively). A possible reason for this difference was the substantial difference in patient numbers between the groups and the highly variable range of these mediators.

The prevalence of keratoconus among patients with VKC ranges from 0.77% to 6% [23]. In the present study, keratoconus was present in 10 patients in Group 3 (50%) and 3 patients in Group 2 (15%) (p=0.018), with an overall incidence of 14%. The higher incidence of keratoconus in this study is attributable to the smaller sample size relative to earlier large-scale studies. VKC is associated with significant ocular morbidity, which thus leads to fewer social interactions and affects the overall quality of life in affected children, including their academic performance. This study also evaluated the quality of life in patients with active disease using a modified Quality of Life Assessment questionnaire. It revealed that 87.5% of patients sometimes had to rub their eyes, 52.5% sometimes used eye drops, and 57.5% sometimes experienced red eyes and a burning sensation during the two weeks preceding evaluation for the study.

This study had certain limitations, such as a smaller sample size for both cases and the control. The method used for tear collection in our research was Schirmer strips, which measure stimulated tears and might interfere with the interpretation of the levels of various inflammatory mediators, which might be lower in stimulated tears because of dilution, as compared to the measurement in basal tears, which are collected using a needle or application of a filter to the eye [23].

## Conclusion

Although various cytokines have been detected in the tears and sera of patients with active disease, compared with controls, with Th2 cytokines more abundant than Th1, none has yet proven to have significant diagnostic or therapeutic value and is not yet classified as a reliable biomarker. Studies with larger sample sizes and improved tear-collection techniques are warranted to ensure a greater volume of unstimulated/basal tears is available to identify a specific biomarker for the disease. This will guide diagnosis and provide customized treatment with specific cytokine blockers, particularly for patients with refractory disease.
